# Comparison of Macintosh Laryngoscope, King Vision^®^, VividTrac^®^, AirAngel Blade^®^, and a Custom-Made 3D-Printed Video Laryngoscope for Difficult and Normal Airways in Mannequins by Novices—A Non-Inferiority Trial

**DOI:** 10.3390/jcm13113213

**Published:** 2024-05-30

**Authors:** Viktor Bacher, Márton Németh, Szilárd Rendeki, Balázs Tornai, Martin Rozanovic, Andrea Pankaczi, János Oláh, József Farkas, Melánia Chikhi, Ádám Schlégl, Péter Maróti, Bálint Nagy

**Affiliations:** 1 Department of Anesthesiology and Intensive Medicine, Medical School, University of Pecs, H-7624 Pecs, Hungary; bacher.viktor@pte.hu (V.B.); nemiabt.pte@tr.pte.hu (M.N.); rendeki.szilard@pte.hu (S.R.); tornai.balazs@pte.hu (B.T.); pankaczi.andrea@pte.hu (A.P.); chikhi.melania@pte.hu (M.C.); nagy.balint@pte.hu (B.N.); 2 Medical Skills Education and Innovation Centre, Medical School, University of Pécs, H-7624 Pecs, Hungary; farkas.jozsef@pte.hu (J.F.); schlegl.adam@pte.hu (Á.S.); 3 Department of Anatomy, Medical School, University of Pécs, H-7624 Pecs, Hungary; 4 Department of Orthopedics, Medical School, University of Pécs, H-7624 Pecs, Hungary; 5 3D Printing & Visualization Centre, Medical School, University of Pécs, H-7624 Pecs, Hungary

**Keywords:** 3D printing, AirAngel Blade^®^, airway management, custom-made device, endotracheal intubation, King Vision^®^, videolaryngoscope, VividTrac^®^

## Abstract

**Background:** Endotracheal intubation (ETI) is a cornerstone of airway management. The gold standard device for ETI is still the direct laryngoscope (DL). However, video laryngoscopes (VLs) are now also widely available and have several proven advantages. The VL technique has been included in the major airway management guidelines. During the COVID-19 pandemic, supply chain disruption has raised demand for 3D-printed medical equipment, including 3D-printed VLs. However, studies on performance are only sparsely available; thus, we aimed to compare 3D-printed VLs to the DL and other VLs made with conventional manufacturing technology. **Methods:** Forty-eight medical students were recruited to serve as novice users. Following brief, standardized training, students executed ETI with the DL, the King Vision^®^ (KV), the VividTrac^®^ (VT), the AirAngel Blade^®^ (AAB), and a custom-made 3D-printed VL (3DVL) on the Laerdal^®^ airway management trainer in normal and difficult airway scenarios. We evaluated the time to and proportion of successful intubation, the best view of the glottis, esophageal intubation, dental trauma, and user satisfaction. **Results:** The KV and VT are proved to be superior (*p* < 0.05) to the DL in both scenarios. The 3DVL’s performance was similar (*p* > 0.05) or significantly better than that of the DL and mainly non-inferior (*p* > 0.05) compared to the KV and VT in both scenarios. Regardless of the scenario, the AAB proved to be inferior (*p* < 0.05) even to the DL in the majority of the variables. The differences between the devices were more pronounced in the difficult airway scenario. The user satisfaction scores were in concordance with the aforementioned performance of the scopes. **Conclusions:** Based upon our results, we cannot recommend the AAB over the DL, KV, or VT. However, as the 3DVL showed, 3D printing indeed can provide useful or even superior VLs, but prior to clinical use, meticulous evaluation might be recommended.

## 1. Introduction

Establishing a definitive airway via endotracheal intubation (ETI) is one of the most important procedures in acute care. Despite the progress which has recently been made, airway manipulation still carries the risk of major complications, including, but not limited to, failed intubation, hypoxia, or even hypoxia-induced cardiac arrest [1,2,3].

To reduce the incidence of these potentially fatal complications, numerous techniques and devices—including video laryngoscopes (VLs)—have been developed to challenge or even replace direct laryngoscopy (DL), which has been the “gold standard” intubation technique for decades [4]. Since the first commercially available VL (GlideScope^®^, 2001), the VL technique has gained ever growing popularity, especially when it comes to difficult airways or severe respiratory instability. Furthermore, in some countries and clinical settings, video laryngoscopy is already recommended to be and has become the new first choice device [5,6,7,8,9].

Studies have shown that VLs in general are suitable for patient care and might be superior—especially in the case of difficult intubation—to the DL regarding success rates and the view of glottis [10,11,12,13]. Based on these findings, the VL has been implemented in all the major airway management guidelines [5,14]. The most important common advantages of VLs are that there is no need for the alignment of the oral–pharyngeal–tracheal axes during laryngoscopy, less pressure and cervical movement are required, and it might provide a faster learning curve [15,16]. However, VLs are heterogenous; each device comes with different features, advantage/disadvantage profiles, and costs. Classifications like channel-bladed vs. non-channel-bladed or standard vs. angulated/hyper-angulated-bladed might help to evaluate the VL’s performance and to give more sophisticated recommendations in different airway scenarios.

During the COVID-19 pandemic, healthcare systems were faced with an overwhelming number of patients and a lack of healthcare professionals and resources, factors which substantially worsened patient outcomes [17,18]. The use of VLs during the pandemic was recommended to decrease disease transmission, the incidence of failed intubations, and time of airway management [19,20].

However, production capacity constraints and supply chain disruption lead to the limited availability of VLs and other healthcare products, which raised the demand for three-dimensional (3D)-printed medical equipment [21,22,23].

Three-dimensional printing is often used in small-scale production and has been proved to be an effective method during the COVID-19 pandemic. Fast, versatile, and cost-effective technology allowed healthcare professionals and engineers to fabricate medical devices like personal protective equipment (PPE), face shields and googles, nasopharyngeal swabs, ventilator valves, and mask connectors [23,24,25]. Additive manufacturing might also support remote medical sites. Wong et al. revealed that it can be used during space missions [26,27] or on remote sites where medical care is limited or prohibited [28,29].

Today, 3D-printed VLs are already commercially available, yet scientific studies on performance are only sparsely published [30,31,32,33,34].

Therefore, our aim was to evaluate how our custom-made 3D-printed VL and a commercially available 3D-printed VL (AirAngel Blade^®^) perform in standardized airway scenarios compared to other laryngoscopes made with conventional manufacturing technology (DL, KingVision^®^, and VividTrac^®^). Primary endpoints were the intubation success rate and time to successful intubation. 

## 2. Materials and Methods

Ethical approval was obtained from the Institutional Scientific and Human Research Ethics Committee of the University of Pécs (5825/2016—approved on the 1 April 2016, 7176/2018—approved on the 6 April 2018) prior to the study. The investigation was carried out at the Medical Skills Lab of the Medical School, University of Pécs, Hungary. Forty-eight voluntary medical students without prior experience in advanced airway management were recruited. All students provided written informed consent prior to participation.

**Figure 1:**
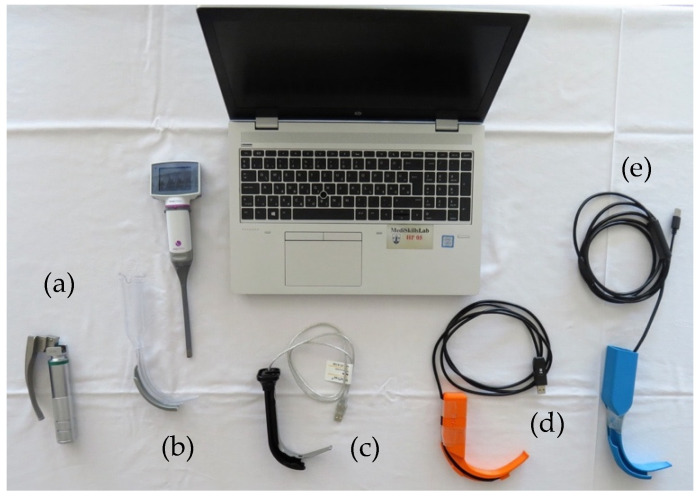
Different laryngoscopes included in the study (from left to right)—(**a**) Macintosh DL with a size 3 blade (KaWe^®^, Asperg, Germany); (**b**) KingVision^®^ VL with a size 3 channeled blade (Ambu^®^, Copenhagen, Denmark); (**c**) VividTrac^®^ VL with an adult channeled blade (Vivid Medical^®^, Palo Alto, CA, USA); (**d**) AirAngel Blade^®^ VL (The Air Angel Project, www.airangelblade.org accessed on 10 April 2024); (**e**) I custom-made 3D-printed VL with a channeled blade (manufactured by the 3D Printing & Visualization Centre of the University of Pécs).

**Figure 2:**
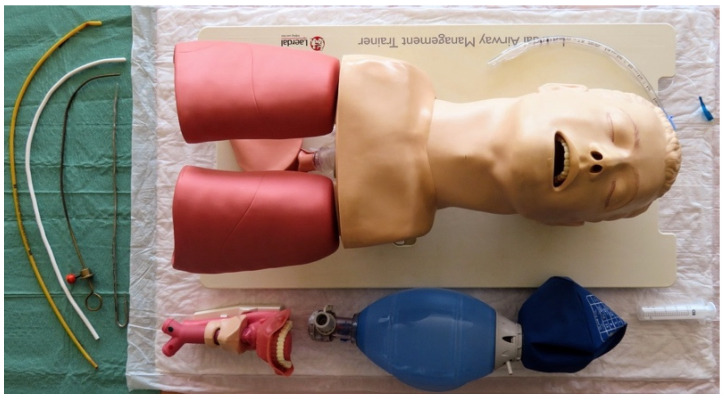
Setting of the separate tables in the study.

We evaluated the following devices in the study (Figure 1): (1) the Macintosh DL with a size 3 blade (DL, KaWe^®^, Asperg, Germany); (2) a custom-made 3D-printed VL with a channeled blade (3DVL, manufactured by the 3D Printing & Visualization Centre of the University of Pécs, Hungary); (3) the KingVision^®^ VL with a size 3 channeled blade (KV, Ambu^®^, Copenhagen, Denmark); (4) the AirAngel Blade^®^ VL (AAB, The Air Angel Project, www.airangelblade.org accessed on 10 April 2024); and (5) the VividTrac^®^ VL with an adult channeled blade (VT, Vivid Medical^®^, Palo Alto, CA, USA). To display real-time videos during the study, except for the KV—which comes with a built-in display—we used a laptop as an external display (Figure 1). The different devices were organized on 5 separate tables, whereby every table was supplied with an airway management trainer (Laerdal^®^, Stavanger, Norway), 2 larynx models (Laerdal^®^, Stavanger, Norway), medical silicone spray (Silomed-Spray^®^, Wesel, Germany), and a bag-valve-mask system besides the laryngoscopes (Figure 2). In the case of the non-channeled scopes (DL and AAB), stylets and bougie were also provided. The production of the 3DVL was carried out using an Ultimaker 3 Extended desktop 3D printer (Ultimaker B.V. Utrecht, the Netherlands) using a 76.3 g neat PLA (polylactic acid) filament. The infill density was 100%; the printing resolution was set to 0.2 mm. For the printing process, a 0.4 mm nozzle was used. The printing speed calibrated to 70 mm/s, while printing temperature was 200 °C and plate temperature was 60 °C, respectively. The laryngoscope was then supplied with an endoscopic camera for imaging.

For the evaluation, we prepared two different airway scenarios. In “Scenario A”, complete reclination of the head was permitted to serve as a normal airway. The difficult airway scenario (“Scenario B”) was created by the manual immobilization of the cervical spine of the mannequin (MILS). MILS is part of the Advanced Trauma Life Support (ATLS) guideline and is often used to simulate difficult airways [35]. A standardized demonstration and a 15 min training on each device for both scenarios were provided to the students by an anesthetist with more than 15 years of clinical experience. Optimization maneuvers like readjustment of the head position, external laryngeal manipulation (ELM), use of stylet/bougie, and Percentage of Glottic Opening (POGO) score estimation were explained and practiced under the supervision of experienced physicians [36,37]. The larynx models served as In important demonstration tool besides the airway trainer during the standardized demonstration and the training session. They were used to explain and demonstrate the correct blade positioning and the way of estimating the best view of the glottis (POGO). The importance and the mechanism of dental injury during ETI were also highlighted. ETI was performed with a standard 7.5 mm internal diameter, cuffed, plastic endotracheal tube (ETT) (Mallinckrodt^®^, Covidien, Dublin, Ireland). The Laerdal^®^ Airway Management Trainer (Laerdal^®^, Stavanger, Norway) was used for demonstrations, trainings, and evaluations during the whole study [16,38,39].

Participants performed ETIs with all devices in both scenarios in a randomly assigned order. As the primary endpoint, we defined successful ETI as the overall success rate (%). Secondary outcomes included the time to successful endotracheal intubation, the time to best glottis view, tube insertion time, the best POGO achieved, the number of intubation attempts, the occurrence of esophageal intubations, the occurrence of dental trauma, and the need for optimization maneuvers. The time elapsed from the tool blade passing the interdental line until the best POGO was achieved (marked as manipulation initiation with the endotracheal tube) was considered laryngoscopy time (LT). The time to successful tracheal intubation was noted as intubation time (IT), and the difference between IT and LT was registered as tube insertion time (TIT). Failed intubation was considered in the case of more than 3 unsuccessful attempts, unrecognized esophageal intubation, or if further attempts were considered futile by the participant. The following attempts were considered failed attempts: attempts that required more than 120 s, esophageal intubation (recognized by the participant), or the device was removed from the oral cavity during the attempt. Importantly, the best POGO achieved was reported as well in the case of the DL by the participant and in the case of the VL by the investigators. In the case of the DL and AAB, stylet and bougie use was also recorded. Considering the most important complications during ETI, the occurrence of esophageal intubation and dental injury was also noted. Following the completion of each scenario, the participants were asked to grade the devices with the help of a 5-point Likert scale based on the ease of technical and physical use (1 = easy and 5 = difficult) and the willingness to reuse (1 = would never use again and 5 = would like to use again) in the relevant scenario, but they were discouraged from ranking the devices overall.

Our data were analyzed using version 22.0 of the Statistical Package for the Social Sciences (SPSS) statistics software (IBM Corporation^®^, Armonk, NY, USA). Continuous and ordinal data are presented as the median and interquartile range (IQR), and the categorical data are presented as raw numbers and as frequencies. Due to non-normal distribution of the data on the Kolmogorov–Smirnov and Shapiro–Wilk tests, non-parametric tests were performed. To assess pair-wise differences between the devices for LT, IT, TIT, POGO score, ease of technical and physical use, and willingness to reuse, we run the Kruskal–Wallis one-way analysis of variance (ANOVA) with Dunn’s post hoc test. To evaluate differences between the devices for the rate of successful tracheal intubation, esophageal intubation, dental injury, and bougie and stylet usage, Chi-square tests were performed. *p* < 0.05 was considered significant.

## 3. Results

We summarized our results in Table 1, Table 2, Table 3 and Table 4. Raw data are presented in Table 1 and Table 3, and *p* values for the comparison of the devices are displayed in Table 2 and Table 4 for Scenario A and B, respectively.

### 3.1. Scenario A

Even though only the VT achieved a 100% first pass success rate in Scenario A, none of the devices showed a significant difference compared to the DL. However, the AAB proved to be significantly inferior compared to the 3DVL and VT in this regard. When it comes to overall intubation time (IT), we found the KV significantly superior and the AAB significantly inferior to the DL, while the 3DVL and VT showed no difference (*p* > 0.05). These differences in IT were mainly due to the differences we noted in tube insertion times (TITs). The shortest TIT in this scenario was related to the KV, while the longest one was related to the AAB (*p* < 0.05). Overall, the longest LTs, TITs, and ITs were all related to AAB. However, all the VLs provided better glottic visualization (POGO) than the DL (*p* < 0.05), while in the VL group, the VT showed the best results (*p* < 0.05). Between the 3DVL and the AAB, we found no significant difference in this regard. Compared to the DL, technical use was easiest by the VT and most difficult by the AAB (*p* < 0.05), while the 3DVL showed significantly better results in this regard compared to the AAB. We noted similar results by physical use, where the VT and KV proved to be superior compared to the DL (*p* < 0.05), and the AAB was non-significantly inferior. The reuse scores were highest by the KV and lowest by the AAB (*p* < 0.05), while the 3DVL was preferred over the AAB in this scenario (*p* < 0.05). We found no significant differences in the incidence of esophageal intubation and the rate of bougie usage. However, dental injuries and the need for stylets were notably higher by non-channeled devices (DL, AAB). 

### 3.2. Scenario B

In the simulated difficult airway scenario, the AAB proved to be inferior regarding the first pass success rate compared to the DL (*p* < 0.05), while other devices showed no difference (*p* > 0.05). Interestingly, the highest first pass success rate was related to the 3DVL in this scenario. All VLs except the AAB proved to be significantly faster regarding overall intubation time (IT) compared to the DL. The fastest ITs were related to the KV and VT, respectively, while the slowest were related to the AAB. The 3DVL was superior to the AAB in this regard as well (*p* < 0.05). Channel-bladed devices (3DVL, KV, and VT) all had significant advantages in terms of tube insertion time (TIT) compared to non-channeled ones. When it comes to laryngoscopy time (LT), only the KV and VT showed significant benefits compared to the DL. All VTs but the AAB improved their POGO scores in comparison with the DL (*p* < 0.05). The technical and physical use scores showed similar results. Except the AAB, all VLs received better scores in this scenario than the DL (*p* < 0.05). However, the operators would prefer to reuse only the KV and the VT over the DL in difficult airways. Between the two 3D-printed VLs, the 3DVL proved to be superior to the AAB in all operator-based subjective parameters. Bougie or stylet use was necessary only by non-channeled devices (DL, AAB). Esophageal intubation occurred only by the DL and KV, but there was no difference between the devices (*p* > 0.05). Interestingly, dental injury was found to be a frequent complication by all scopes. We experienced no difference compared to the DL in this regard (*p* > 0.05). 

## 4. Discussion

Using advanced airway management techniques, especially ETI, remains a critical skill in various medical fields; it can be challenging to master and requires constant practice to maintain. The importance of ETI is underlined by the fact that the inability to secure the airway is one of the leading causes of anesthesia-related complications and might have life-threatening consequences [15,40,41]. In light of the events of recent years due to the COVID-19 pandemic, both the technical and personnel aspects of airway management have had to face new challenges. The global shortage of material and human resources has led to an increasing demand for alternative devices, such as VLs in airway management [19,21]. This shortage also encouraged engineers and physicians to create improvised and custom-made devices with additive manufacturing techniques like 3D printing. Despite the previous promising results in various settings, many questions remained unclear, especially regarding the utility of the latest and scientifically sparsely tested 3D-printed VLs [6,7,10,11,12,13,15,16,38,39,41,42]. Therefore, we aimed to evaluate two 3D-printed VLs (AAB, 3DVL) and compare them to already well-tested conventional devices (DL, KV, VT). 

Previous studies have already compared the DL to VLs in different simulated scenarios. The study of Kluj. et al. compared three non-channeled VLs and the Macintosh DL in a simulated environment where PPE-wearing novice users had to execute the intubation, and they could not prove that VLs presented a significant advantage regarding first pass success rates and intubation times [43]. A recent study of Muhamed et al.—evaluating a similar number of novices as our study—found that the evaluated VL performed better in terms of time-related metrics, glottic visualization, and incidence of dental injury, but it showed similar results when it came to success rates and ease of use [44]. The GlideScope^®^ was also compared to Macintosh DL in three simulated airway settings of a normal airway, neck immobility, and tongue edema, where the VL improved the view of vocal cords, increased intubation success rates, and decreased the time to successful intubation and time to best glottis view in the tongue-edema-related difficult airway setting [45]. A recent study of La Via et al. comparing two VLs (GlideScope^®^, McGrath^®^) with DL and with a combined laryngo-bronchoscopy technique found that anesthesia residents achieved best success rates and intubation times with DL, which they partially explained with their prior experience with the DL technique [46].

Apart from the KV in the difficult airway scenario, the overall success rate of the evaluated devices was unanimously over 90%. The best overall and first-attempt success rates were achieved by the 3DVL and the VT in both scenarios, but these results did not prove to be superior to the DL in our settings. However, regarding the first-pass success rate, the AAB was found to be significantly inferior to the 3DVL and similar or worse to the DL, KV, and VT in both scenarios. The relatively poor performance of AAB is in concordance with Ataman et al., who recently found a more than 40% lower first-pass success rate by the AAB compared to GlideScope^®^ in simulated normal and difficult airways [31]. Since the 3DVL is our custom-made device, its promising results could not be directly compared to previous studies, but it supports the hypothesis that 3D printing in general could deliver clinically useful VLs [30,33]. Lambert et al. 3D printed and tested a channel-bladed “Tansen” VL, which has similar features to our 3DVL. They also obtained excellent results. Their “Tansen” VL was superior to the DL and non-inferior to Pentax-AWS in terms of success rate, grade of view, and time to intubation [30]. Anyway, both the 3DVL and VT are channel-bladed and have an external display, which seems to be a superior combination in our settings.

For every device, IT was consistently under 20 and 25 s in Scenario A and B, respectively, which is in line with our previous study [41]. In both scenarios, participants accomplished the shortest IT with the KV. Since all other VLs operated with an externally attached display system which was placed further from the mannequin`s head, we anticipated that the built-in display`s proximity to the manipulation site might result in improved eye–hand coordination, leading to a shorter manipulation time. When it comes to 3D-printed devices, the 3DVL proved to be significantly superior to the AAB regarding IT in both scenarios. This result mainly comes from the shorter tube insertion time by the 3DVL, which might be explained by its tube-guiding channel. However, in general, the role of the tube-guiding channel in terms of success rates and intubation times is still a matter of intense debate [47,48,49]. While the 3DVL was found to be non-inferior or, to some extent, even superior to the DL in IT, the AAB mainly proved inferior in this regard. This result of the AAB is in line with Ataman’s study, where the AAB’s intubation times were only comparable in difficult airways but far more inferior in normal airways [31].

In concordance with a previous study from Kaplan Et. al., the glottic visualization (POGO) with VLs was, overall, significantly better than with DL [50]. The only exception was the AAB in this regard, which provided similar POGO scores to the DL in the difficult airway scenario. Our custom-made 3D-printed device (3DVL) proved to be superior to the DL and non-inferior to the AAB in both scenarios.

The novice operators found the KV, VT, and 3DVL easiest to use, especially in the more challenging difficult airway scenario. In line with its performance, the non-channel-bladed AAB ended up being the least preferred VL of our study. These results are consistent with those of previous studies [39,41,51].

Regardless of the intubation device, the incidence of the esophageal intubations was relatively low, while dental injury happened with a high frequency, especially in Scenario B. It is proven that the VL technique and the lack of experience might increase the incidence of dental trauma during laryngoscopy. Furthermore, the applied force on incisors is significantly affected by the blade type [41,52,53].

Considering all aspects of our study, the 3DVL was proved to be superior to the DL and performed in a comparable way to the conventionally manufactured VLs (KV and VT) in both scenarios. However, the first commercially available 3D-printed VL (AAB) was found to be mainly inferior in our study not only to the VLs but also to the DL as well. We speculated that the cause of this might be the different blade type, especially since it is proven that novice/less-experienced users prefer channeled blades over non-channeled ones [54]. However, the AAB was inferior compared to even other non-channeled VL devices, like the GlideScope^®^, reporting lower first attempt success rates and longer intubation times [31]. Furthermore, regardless of the airway scenario, successful intubation with the AAB was almost only possible with the help of a stylet, which is in line with experts’ opinions and previous recommendations on stylet/bougie use by non-channel-bladed devices [53,54]. Recent studies on the DL vs. VL technique among novice users have presented inconsistent findings on the superiority of one technique over the other, but improved time metrics and glottic visualization with VLs have been observed, especially in the difficult airway scenario, which is in concordance with our study. Our findings also support previously described similar success rates of the two techniques and the questionable advantage of non-channeled devices for novice operators [43,44,45,46].

Since our study was a monocentric mannequin study performed in a simulation environment evaluating only novice users with no previous experience in endotracheal intubation, the degree of the direct transferability of our findings into clinical practice remains unclear. The preclinical nature of our study warrants further, more detailed investigations in clinical settings, including more experienced intubators and real patients. Further factors, like the short time gap between training and evaluation, the standardized setting, and the anatomical fidelity of the airway trainer, underline this as well. A further limiting factor is presented in the assessment of dental trauma, where the extent of trauma was not measured.

## 5. Conclusions

Based upon our results, we cannot recommend AirAngle Blade^®^ over direct laryngoscope, King Vision^®^, or VividTrac^®^. However, 3D printing indeed can provide useful or even superior video laryngoscopes, but prior to clinical use, meticulous evaluation is recommended in various airway scenarios to select useful and suitable devices. Even custom-designed and -made 3D-printed scopes—like the 3DVL in our study—might surpass the “gold standard” or modern, commercially available devices.

## 6. Limitations of the Study

Since our study was a monocentric mannequin study performed in a simulation environment evaluating only novice users with no previous experience in endotracheal intubation, the degree of the direct transferability of our findings into clinical practice remains unclear. The preclinical nature of our study warrants further, more detailed investigations in clinical settings, including more experienced intubators and real patients. Further factors, like the short time gap between training and evaluation, the standardized setting, and the anatomical fidelity of the airway trainer, underline this as well. A further limiting factor is presented in the assessment of dental trauma, where the extent of trauma was not measured.

## Figures and Tables

**Table 1:** Data for Scenario A. Data are reported as median [IQR] or as numbers (*n*). Significant difference was considered *p* < 0.05.

Scenario A	DL (*n* = 48)	3DVL (*n* = 48)	KV (*n* = 48)	AAB (*n* = 48)	VT (*n* = 48)
Number of attempts (*n*, 1/2/3/not successful)	43/2/1/2	46/2/0/0 ¶	45/2/0/1	38/9/0/1 §#	48/0/0/0 ¶
Laryngoscopy time (s)	8.12 [6.79–10.46]	7.44 [5.82–9.74]	7.1 [5.55–9.37] ¶	8.95 [7.76–12.1] †§	7.14 [5.65–9.02] ¶
Tube insertion time (s)	5.46 [3.67–8.44] ¶†	6.12 [3.25–10.25] †	3.5 [2.21–5.45] *¶#	8.64 [5.1–16.1] *†§	5.62 [3.1–7.1] ¶
Intubation time (s)	14.12 [11.69–18.22] ¶†	14.23 [9.97–20.6] †¶	11.12 [8.8–12.9] *¶#	18.68 [14.3–25.1] *†§#	12.25 [9.98–15.29] ¶
POGO (%)	70 [50, 80] †§#¶	90 [85–95] *§	95 [80–100] *§	90 [70–95] *§	100 [95–100] *#†¶
Ease of technical use (1–5)	3 [2–3] §¶	2 [2–3] §¶	2 [1–2] ¶	3 [3–4] *#†§	1 [1–2] *#¶
Ease of physical use (1–5)	2 [2–3] †§	2 [1–3] ¶	1 [1–2] *¶	3 [2–3] #†§	1 [1–2] *¶
Willingness of reuse (1–5)	4 [3–5] †¶	4 [3–5] †¶§	5 [4–5] *#¶	3 [2–3] *#†§	5 [4–5] #¶
Use of bougie (*n*)	1	0	0	2	0
Use of stylet (*n*)	17 †§#¶	0 *¶	0 *¶	38 *†§#	0 *¶
Dental injury (*n*)	17 †§#	2 *¶	1 *¶	9 §†#	1 *¶
Esophageal intubation (*n*)	3	0	0	0	0

* Significant difference compared to DL; # significant difference compared to 3DVL; † significant difference compared to KV; ¶ significant difference compared to AAB; § significant difference compared to VT; DL: direct laryngoscope (Macintosh); 3DVL: custom-made 3D-printed video laryngoscope; KV: King Vision^®^; AAB: AirAngel Blade^®^; VT: VividTrac^®^. POGO: Percent of Glottic Opening.

**Table 2:** *p* values for pairwise comparison of the evaluated devices in Scenario A.

Scenario A	DL vs. 3DVL	DL vs. KV	DL vs. AAB	DL vs. VT	3DVL vs. KV	3DVL vs. AAB	3DVL vs. VT	KV vs. AAB	KV vs. VT	AAB vs. VT
Number of attempts (*n*, 1/2/3/not successful)	NS	NS	NS	NS	NS	*p* < 0.05	NS	NS	NS	*p* < 0.05
Laryngoscopy time (s)	NS	NS	NS	NS	NS	NS	NS	*p* < 0.05	NS	*p* < 0.05
Tube insertion time (s)	NS	*p* < 0.05	*p* < 0.05	NS	*p* < 0.05	NS	NS	*p* < 0.05	NS	*p* < 0.05
Intubation time (s)	NS	*p* < 0.05	*p* < 0.05	NS	*p* < 0.05	*p* < 0.05	NS	*p* < 0.05	NS	*p* < 0.05
POGO (%)	*p* < 0.05	*p* < 0.05	*p* < 0.05	*p* < 0.05	NS	NS	*p* < 0.05	NS	*p* < 0.05	*p* < 0.05
Ease of technical use (1–5)	NS	NS	*p* < 0.05	*p* < 0.05	NS	*p* < 0.05	*p* < 0.05	*p* < 0.05	NS	*p* < 0.05
Ease of physical use (1–5)	NS	*p* < 0.05	NS	*p* < 0.05	NS	*p* < 0.05	NS	*p* < 0.05	NS	*p* < 0.05
Willingness of reuse (1–5)	NS	*p* < 0.05	*p* < 0.05	NS	*p* < 0.05	*p* < 0.05	*p* < 0.05	*p* < 0.05	NS	*p* < 0.05
Use of bougie (*n*)	NS	NS	NS	NS	NS	NS	NS	NS	NS	NS
Use of stylet (*n*)	*p* < 0.05	*p* < 0.05	*p* < 0.05	*p* < 0.05	NS	*p* < 0.05	NS	*p* < 0.05	NS	*p* < 0.05
Dental injury (*n*)	*p* < 0.05	*p* < 0.05	NS	*p* < 0.05	NS	*p* < 0.05	NS	*p* < 0.05	NS	*p* < 0.05
Esophageal intubation (*n*)	NS	NS	NS	NS	NS	NS	NS	NS	NS	NS

DL: direct laryngoscope (Macintosh); 3DVL: custom-made 3D-printed video laryngoscope; KV: King Vision^®^; AAB: AirAngel Blade^®^; VT: VividTrac^®^. POGO: Percent of Glottic Opening; NS: not significant; *p* < 0.05: *p* value smaller than 0.05—significant difference.

**Table 3:** Data for Scenario B. Data are reported as median [IQR] or as numbers (*n*). Significant difference was considered *p* < 0.05.

Scenario B	DL (*n* = 48)	3DVL (*n* = 48)	KV (*n* = 48)	AAB (*n* = 48)	VT (*n* = 48)
Number of attempts (*n*, 1/2/3/not successful)	43/1/1/3 ¶	46/2/0/0 †¶	37/5/1/5 #	33/11/1/3 *#§	45/2/1/0 ¶
Laryngoscopy time (s)	12.18 [9.03–18] †§	10.2 [7.7–13.3]	8.6 [7–14.5] *¶	12.8 [7.37–11.7] †§	8.7 [6.9–10.3] *¶
Tube insertion time (s)	6.3 [3.8–11.1] #†§	2.6 [1.5–4.2] *¶	3.4 [2.1–5.2] *¶	8.1 [4.3–11.4] #†§	4.2 [2.4–6.7] *¶
Intubation time (s)	19.5 [14.9–26.1] #†§	14.5 [9.8–16.8] *¶	13 [10.3–20.1] *¶	21.2 [16.5–26.7] #†§	13.3 [10.3–18.1] *¶
POGO (%)	50 [32.5–60] #†§	65 [50–84] *¶	70 [50–80] *¶	50 [30–65] #†§	72.5 [60–90] *¶
Ease of technical use (1–5)	3 [2–4] #†§	2 [2–3] *¶	2 [2–3] *¶	3 [3–4] #†§	2 [2–3] *¶
Ease of physical use (1–5)	3 [2–4] #†§	2 [1–3] *¶	2 [1–3] *¶	3 [3–4] #†§	2 [1–2] *¶
Willingness of reuse (1–5)	3 [2–4] †§	4 [3–5] ¶	5 [4–5] *¶	3 [2–3] #†§	4 [4–5] *¶
Use of bougie (*n*)	6 #†§	0 *	0 *	1	0 *
Use of stylet (*n*)	21 #†¶§	0 *¶	0 *¶	41 *#†§	0 *¶
Dental injury (*n*)	29	26 ¶	26 ¶	35 #†	30
Esophageal intubation (*n*)	3	0	2	0	0

* Significant difference compared to DL; # significant difference compared to 3DVL; † significant difference compared to KV; ¶ significant difference compared to AAB; § significant difference compared to VT; DL: direct laryngoscope (Macintosh); 3DVL: custom-made 3D-printed video laryngoscope; KV: King Vision^®^; AAB: AirAngel Blade^®^; VT: VividTrac^®^. POGO: Percent of Glottic Opening.

**Table 4:** *p* values for pairwise comparison of the evaluated devices in Scenario B.

Scenario B	DL vs. 3DVL	DL vs. KV	DL vs. AAB	DL vs. VT	3DVL vs. KV	3DVL vs. AAB	3DVL vs. VT	KV vs. AAB	KV vs. VT	AAB vs. VT
Number of attempts (*n*, 1/2/3/not successful)	NS	NS	*p* < 0.05	NS	*p* < 0.05	*p* < 0.05	NS	NS	NS	*p* < 0.05
Laryngoscopy time (s)	NS	*p* < 0.05	NS	*p* < 0.05	NS	NS	NS	*p* < 0.05	NS	*p* < 0.05
Tube insertion time (s)	*p* < 0.05	*p* < 0.05	NS	*p* < 0.05	NS	*p* < 0.05	NS	*p* < 0.05	NS	*p* < 0.05
Intubation time (s)	*p* < 0.05	*p* < 0.05	NS	*p* < 0.05	NS	*p* < 0.05	NS	*p* < 0.05	NS	*p* < 0.05
POGO (%)	*p* < 0.05	*p* < 0.05	NS	*p* < 0.05	NS	*p* < 0.05	NS	*p* < 0.05	NS	*p* < 0.05
Ease of technical use (1–5)	*p* < 0.05	*p* < 0.05	NS	*p* < 0.05	NS	*p* < 0.05	NS	*p* < 0.05	NS	*p* < 0.05
Ease of physical use (1–5)	*p* < 0.05	*p* < 0.05	NS	*p* < 0.05	NS	*p* < 0.05	NS	*p* < 0.05	NS	*p* < 0.05
Willingness of reuse (1–5)	NS	*p* < 0.05	NS	*p* < 0.05	NS	*p* < 0.05	NS	*p* < 0.05	NS	*p* < 0.05
Use of bougie (*n*)	*p* < 0.05	*p* < 0.05	NS	*p* < 0.05	NS	NS	NS	NS	NS	NS
Use of stylet (*n*)	*p* < 0.05	*p* < 0.05	*p* < 0.05	*p* < 0.05	NS	*p* < 0.05	NS	*p* < 0.05	NS	*p* < 0.05
Dental injury (*n*)	NS	NS	NS	NS	NS	*p* < 0.05	NS	*p* < 0.05	NS	NS
Esophageal intubation (*n*)	NS	NS	NS	NS	NS	NS	NS	NS	NS	NS

DL: direct laryngoscope (Macintosh); 3DVL: custom-made 3D-printed video laryngoscope; KV: King Vision^®^; AAB: AirAngel Blade^®^; VT: VividTrac^®^. POGO: Percent of Glottic Opening; NS: not significant; *p* < 0.05: *p* value smaller than 0.05—significant difference.

## Data Availability

The original contributions presented in the study are included in the article, further inquiries can be directed to the corresponding author/s.
